# Degradation of aflatoxin B_1_ from naturally contaminated maize using the edible fungus *Pleurotus ostreatus*

**DOI:** 10.1186/s13568-017-0415-0

**Published:** 2017-06-02

**Authors:** Lauren W. Jackson, Barry M. Pryor

**Affiliations:** 1 School of Plant Sciences, University of Arizona, 1140 E South Campus Drive, P. O. Box 210036, Tucson, AZ 85721 USA; 2 Department of Plant Pathology, University of Minnesota, 1991 Upper Buford Circle, St. Paul, MN 55108 USA

**Keywords:** Aflatoxin, *Pleurotus ostreatus*, Solid-state fermentation, Biodegradation

## Abstract

Aflatoxins are highly carcinogenic secondary metabolites that can contaminate approximately 25% of crops and that cause or exacerbate multiple adverse health conditions, especially in Sub-Saharan Africa and South and Southeast Asia. Regulation and decontamination of aflatoxins in high exposure areas is lacking. Biological detoxification methods are promising because they are assumed to be cheaper and more environmentally friendly compared to chemical alternatives. White-rot fungi produce non-specific enzymes that are known to degrade aflatoxin in in situ and ex situ experiments. The aims of this study were to (1) decontaminate aflatoxin B_1_ (AFB_1_) in naturally contaminated maize with the edible, white-rot fungus *Pleurotus ostreatus* (oyster mushroom) using a solid-state fermentation system that followed standard cultivation techniques, and to (2) and to assess the risk of mutagenicity in the resulting breakdown products and mushrooms. Vegetative growth and yield characteristics of *P. ostreatus* were not inhibited by the presence of AFB_1_. AFB_1_ was degraded by up to 94% by the Blue strain. No aflatoxin could be detected in *P. ostreatus* mushrooms produced from AFB_1_-contaminated maize. Moreover, the mutagenicity of breakdown products from the maize substrate, and reversion of breakdown products to the parent compound, were minimal. These results suggest that *P. ostreatus* significantly degrades AFB_1_ in naturally contaminated maize under standard cultivation techniques to levels that are acceptable for some livestock fodder, and that using *P. ostreatus* to bioconvert crops into mushrooms can reduce AFB_1_-related losses.

## Introduction

Aflatoxins are highly carcinogenic secondary metabolites produced by several members of *Aspergillus* section *Flavi* that contaminate an array of important food crops (Cotty et al. 1994). These mycotoxins are responsible for causing adverse health conditions in humans and other animals such as growth retardation, immune suppression, hepatocellular carcinoma, and death (Kensler et al. 2011). Aflatoxins are tightly regulated in the U.S. and European Union (EU), where the maximum allowable aflatoxin concentration in foods intended for human consumption range from 4 to 20 ng g^−1^ (Henry et al. 1999). The maximum contamination level of feed intended for non-dairy livestock in the U.S. is 300 ng g^−1^ (Kensler et al. 2011). Regulation of mycotoxins is lacking in many developing nations where aflatoxin-related health problems are most severe, and approximately 4.5 billion people are chronically exposed (Williams et al. 2004). Efforts to control aflatoxin exposure in these areas are complicated for several reasons: (1) proper infrastructure is not in place; (2) testing and chemical decontamination are not economically feasible for many producers; and (3) many producers in high-exposure areas are subsistence farmers (Williams et al. 2004; Cotty et al. 2008).

Biological decontamination methods present possible advantages to chemical processes because they are less likely to be detrimental to the environment and are assumed to be more cost-effective (Guan et al. 2011). Methods involving the use of fungi that secrete non-selective, ligninolytic enzymes are among the most promising biological decontamination strategies studied thus far (Kim et al. 2017). Motomura et al. (2003) isolated an unidentified enzyme from *Pleurotus ostreatus* that reduced the fluorescence of AFB_1_ and attributed this to the disruption of its lactone ring that plays an important role in carcinogenicity. Alberts et al. (2009) later showed that supernatant from *P. ostreatus* degraded AFB_1_ by up to 76% and that degradation efficiency was strongly correlated with the activity level of pure laccase. Wang et al. (2011) and Yehia (2014) used manganese peroxidases from *Phanerochaete sordida* and *P. ostreatus*, respectively, to degrade AFB_1_ in ex situ experiments. In a microcosm study, Das et al. (2014) demonstrated enhanced degradation of AFB_1_ in rice straw by *P. ostreatus* in the presence of certain surfactants and metal salts, and identified several potential breakdown products. Two strains of *P. ostreatus* were also used to degrade AFB_1_ in a co-cultivation experiment with *Aspergillus flavus* on rice straw revealing that one strain demonstrated superior degradation efficiency (Das et al. 2015).

To date, the effect of AFB_1_ on growth characteristics that are important for *P. ostreatus* mushroom production such as colonization rates, mushroom development and yield have not been reported. It is also not known if aflatoxins can accumulate in mushrooms produced from aflatoxin-contaminated substrates. Furthermore, the potential for AFB_1_ breakdown products to revert under simulated conditions of the human stomach, a known phenomenon that has been evaluated for chemical decontamination technologies (Weng et al. 1994), has not been tested in any biological decontamination studies. In the present study, three commercial strains of *P. ostreatus* (N001, Pearl and Blue) were grown on maize that was contaminated at three concentrations (25, 250 and 2500 ng g^−1^) of AFB_1_ in mesocosms using standard mushroom cultivation techniques. The aims of this study were to (1) evaluate the growth characteristics of *P. ostreatus* cultivated in the presence of AFB_1_, (2) test for residual AFB_1_ in substrates and *P. ostreatus* mushrooms, and (3) asses the mutagenicity risk of breakdown products.

## Materials and methods

### Fungal strains, materials, and culture conditions

*Pleurotus ostreatus* var. *columbinus* (“Blue Oyster”) and *P. ostreatus* (“Pearl Oyster”) were obtained from Fungi Perfecti, LLC (Olympia, WA); and *P. ostreatus* (N001) was obtained from the Spanish Type Culture Collection (CECT; Paterna, Spain). All three strains are commercial and dikaryotic. Cultures were maintained in water vouchers at 4 °C until required for experiments. Cultures were grown out on malt yeast peptone agar (MYPA) consisting of 7 g L^−1^ malt extract, 0.5 g L^−1^ yeast extract, 1 g L^−1^ peptone and 15 g L^−1^ agar at room temperature (~23 °C) for 10 days in 100 mm × 15 mm Petri dishes before use for substrate inoculation. *A. flavus* AF13 (ATCC 96044) was provided by Dr. Peter Cotty, USDA–ARS, School of Plant Sciences, University of Arizona. AF13 was grown for spore production on 5-2 Agar (Cotty and Misaghi 1984) consisting of 20 g L^−1^ Bacto™ agar (Becton, Dickinson and Company, Franklin Lakes, NJ) and 50 mL L^−1^ V8™ Juice (CSC Brands, L.P., Camden, NJ) adjusted to pH 6.0 for 7 days at 31 °C. Conidia were harvested from AF13 cultures after 7 days of growth, dispensed into sterile water, enumerated using an Orbeco Hellige TB300 IR Turbidimeter (Orbeco Hellige Inc., Sarasota, FL), diluted to 1.5 × 10^−7^ conidia mL^−1^, and used on the same day to inoculate maize for aflatoxin production. Nature’s Match™ whole corn (maize) (Land O’ Lakes Purina Feed LLC, Shoreview, Minnesota) was purchased from a local feed store. Maize was homogenized and stored at room temperature until use. Three 50 g subsamples of maize were taken to establish baseline levels of aflatoxin contamination. Baseline moisture content (MC) was measured using a Mettler Toledo HB43 Halogen Moisture Analyzer (Mettler-Toledo International Inc., Columbus, OH).

### Aflatoxin production

Maize was added to quart size mason jars (150 g jar^−1^), adjusted to 20% MC, sterilized, and inoculated with 10^5^ AF13 conidia g^−1^. Inoculated maize was adjusted to 30% MC and mixed to distribute spores evenly. Jars were fitted with pre-sterilized lids containing seven ½^”^ holes and synthetic filter discs, and incubated at 31 °C for 7 days. Colonized jars were autoclaved for 30 min at 121 °C and 15 psi to kill the AF13 culture. Aflatoxin-contaminated maize was dried to completeness in a horizontal airflow oven (VWR International LLC, Radnor, PA) set at 45 °C for 7 days. The dried and contaminated maize was homogenized using a Bunn^®^ G3 Coffee Mill (Bunn, Springfield, Illinois), quantitated, and stored at −20 °C until use for substrate preparation.

### Aflatoxin quantitation

Baseline aflatoxin levels and 10× concentrated mushroom samples were quantitated using the Neogen^®^ Reveal^®^ Q+ test kit and AccuScan Pro Reader (Neogen Corp., Lansing, MI) per manufacturer instructions. AFB_1_ from other samples was quantitated via thin layer chromatography (TLC) by fluorometric measurement with a CAMAG TLC Scanner 3 densitometer (CAMAG Scientific, Inc., Wilmington, NC) (Stoloff and Scott 1984; Pons et al. 1966). Limits of detection for this method were established using a ½ log dilution series of AFB_1_ (Sigma-Aldrich Corp., St. Louis, MO). All maize samples that fell below the detection limit after decontamination were concentrated 100× and re-quantitated fluorometrically.

### Substrate preparation

Maize that was uncontaminated (<5 ng g^−1^, but assumed to be 0 ng g^−1^ for calculations and figures herein) or contaminated (25, 250, or 2500 ng g^−1^) with aflatoxin was used for controls or as substrate for *P. ostreatus* growth and mushroom production. Maize samples were adjusted to 45% MC, and 100 g each were added to 4″ × 3″ × 18″ high density polypropylene bags containing a 0.5 µm filter patch. The bags containing maize samples were autoclaved at 121 °C and 15 psi for 60 min. Samples were either mock inoculated with ten 6 mm diameter sterile agar plugs or inoculated with ten 6 mm agar plugs of N001, Pearl, or Blue strains, respectively. Thus, there were 16 sample types including treatment groups and positive and negative controls, with 5 biological replicates used for each sample type (N = 80).

### Colonization and fructification

Samples were incubated at 25 °C for 21 days, and subsequently placed in a fruiting chamber at 90–99% relative humidity, <600 ppm CO_2_, and 23 °C until mushrooms were ready for harvest. Radial mycelial growth could not be measured because the 100 g maize samples were in three-dimensional substrate blocks, so growth was checked daily and the number of days required to completely colonize the substrate was recorded. Bags inoculated with *P. ostreatus* were sliced upon being placed in the fruiting chamber. Bags that were mock inoculated were not sliced to prevent contamination, but were kept in the fruiting chamber until all mushrooms were harvested. The number of days required for mushrooms to mature was recorded to evaluate potential inhibition. Fresh weights of mushrooms were measured immediately after being harvested and data were expressed as biological efficiency (%BE = fresh mushroom weight/dry substrate weight × 100). Processed samples and mushrooms were stored at −20 °C until they were removed to be dried to completeness at 45 °C, and were again stored at −20 °C until analyzed.

### Acid reversion assay

A subset of 10 g substrate samples from all control and treatment groups originally contaminated at 2500 ng g^−1^ AFB_1_, in addition to uncontaminated controls, were subjected to conditions approximating physiological conditions of the human stomach (37 °C and pH 2) for 2 h by addition of 20 mL 0.2 M HCl. Samples were dried in the dark for 48 h at 45 °C and −10 kPa in a vacuum oven, and re-quantitated fluorometrically to assess the extent of chemical reversion to AFB_1_. Data were expressed as percent degradation relative to control samples before and after being subjected to acid reversion conditions.

### *Salmonella typhimurium* (Ames) mutagenicity assay

The *Salmonella* (Ames) mutagenicity assay with metabolic activation was used as a proxy to measure the carcinogenicity of degradation products (Ames et al. 1975). Tester strain TA1535 (Molecular Toxicology Inc., Boone, NC) was used because it has a –G–G–G– DNA target and is susceptible to the same base substitutions that AFB_1_ induces (Foster et al. 1983). A subset of samples from *P. ostreatus*-treated and mock inoculated groups originally contaminated at 2500 ng g^−1^AFB_1_, in addition to uncontaminated control substrate samples, were used for this assay. A serial dilution of pure AFB_1_ in 70% MeOH ranging from 0 to 10,000 ng g^−1^ was used as a reference. Controls containing only 70% MeOH and S-9 metabolic mix without aflatoxin were also used. Samples from both runs of the degradation experiment were assayed concurrently and compared to a single set of control samples. Three biological and three technical replicates were used for each treatment and control group. Colonies were enumerated using OpenCFU software (Geissmann 2013). Data are given as the number of revertant colony forming units (CFUs).

### Data analyses

All experiments were duplicated unless otherwise noted. Results from duplicated experiments are only shown if they were inconsistent with the initial results. Normality of data was assessed using the Shapiro–Wilk goodness-of-fit test. One-way analysis of variance (ANOVA) tests were conducted for the number of days until colonization, number of days required for mushroom formation, %BE, degradation percentage, and acid reversion data. If significant differences were detected by ANOVA at the 95% confidence level, Tukey’s HSD was used to compare means and generate connecting letter reports. Statistical analyses were conducted using JMP Statistical Discovery Software v10.0 (SAS, Cary, NC).

## Results

### Growth characteristics of *P. ostreatus* cultivated on AFB_1_-contaminated maize

The presence of aflatoxin did not appear to inhibit the vegetative growth of any *P. ostreatus* strains (Table 1). Mushrooms produced by N001 cultivated on maize with the greatest concentration (2500 ng g^−1^) of AFB_1-_ matured significantly faster than those grown from maize contaminated at 250 ng g^−1^, but did not differ from those grown on maize contaminated at 0 or 25 ng g^−1^ (Table 1). Differences between the times required for mushrooms to mature were not observed for the other strains regardless of the concentration of AFB_1_ in their respective substrates (Table 1). Importantly, mushroom yield (%BE) was not reduced by increased concentrations of AFB_1_ in the substrate for any of the strains tested (Table 1).

**Table 1:** Growth characteristics of *P. ostreatus* cultivated on AFB1-contaminated maize

Growth characteristic	AFB_1_ ng g^−1^	Strain			
		Mock	N001	Pearl	Blue
No. days to colonize	0	N/A	10.0 ± 0.32^a^	9.4 ± 0.25^a^	11.0 ± 0.63^a^
	25	N/A	11.2 ± 0.49^a^	10.0 ± 0.32^a^	9.8 ± 0.2^a^
	250	N/A	11.6 ± 0.98^a^	11.4 ± 0.98^a^	10.0 ± 0.0^a^
	2500	N/A	11.2 ± 0.58^a^	10.4 ± 0.4^a^	10.0 ± 0.0^a^
No. days to maturity	0	N/A	18.6 ± 3.2^ab^	14.4 ± 0.68^a^	27.8 ± 2.54^a^
	25	N/A	23.6 ± 4.19^ab^	14.2 ± 0.74^a^	28.6 ± 1.12^a^
	250	N/A	25.8 ± 5.47^a^	13.6 ± 0.25^a^	24.6 ± 2.29^a^
	2500	N/A	10 ± 0.95^b^	16.0 ± 0.63^a^	27.0 ± 2.53^a^
%BE	0	N/A	21.8 ± 2.94^a^	26.3 ± 1.46^a^	23.5 ± 3.45^a^
	25	N/A	19.2 ± 2.1^a^	23.7 ± 1.4^a^	26.1 ± 3.73^a^
	250	N/A	21.4 ± 4.51^a^	24.5 ± 3.41^a^	19.7 ± 3.44^a^
	2500	N/A	26.7 ± 6.19^a^	27.5 ± 3.68^a^	24.7 ± 2.43^a^

Values represent the mean ± standard error. Values not followed by the* same letter* within columns of the same category are significantly different (*p* < 0.05) by Tukey’s HSD

### *P. ostreatus* degradation of AFB_1_ in naturally contaminated maize

**Fig. 1:**
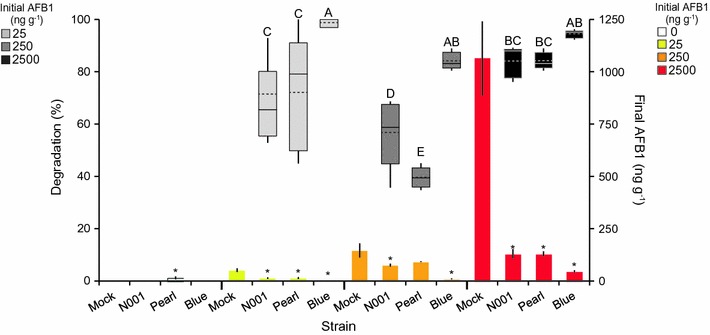
Degradation of AFB_1_ by *P. ostreatus.* Box plots show the percentage of AFB_1_ degraded (*left axis*) by *P. ostreatus* strains grown on uncontaminated (0 ng/g) or contaminated maize at 25, 250 and 2500 ng g^−1^ AFB_1_, respectively. *Bars* show raw AFB_1_ concentrations (*right axis*) of treated (N001, Pearl and Blue) or untreated (mock) samples. *Whiskers on box plots* show the minimum and maximum values and means are indicated by *dashed lines*. Box plot values not connected by the *same letter* are significantly different (*p* ≤ 0.05) by Tukey’s HSD. *Whiskers on bars* indicate standard errors. *Asterisks above bars* show significant difference (*p* ≤ 0.05) by Tukey’s HSD of treated values compared to controls of the same initial AFB_1_ concentration

*Pleurotus ostreatus* significantly degraded aflatoxin in nearly all strain-AFB_1_ concentration combinations (Fig. 1). Only Pearl at 250 ng g^−1^ did not demonstrate significant AFB_1_ degradation compared to control samples (note that AFB_1_ was naturally attenuated in all control samples over the approximately 6 week study period). Treatment with the Blue strain resulted in >90% degradation in samples with an initial AFB_1_ concentration of 2500 ng g^−1^, and the Blue strain demonstrated the smallest variance of AFB_1_ degradation across different concentrations (Fig. 1). The N001 and Pearl strains were less consistent, but still degraded AFB_1_ in maize that had an initial concentration of 2500 ng g^−1^ by >80% (Fig. 1).

### Revertion of AFB_1_ breakdown products

Significant reversion of AFB_1_ under acidic conditions (pH 2) at 37 °C occurred for degradation products resulting from treatment with N001 and Blue in the first run of a duplicated experiment (Table 2). In these cases, the average degradation percentage resulting from treatment with N001 and Blue fell from 91.5 to 80.8%, and 94.3 to 88.8%, respectively (Table 2). Breakdown products did not significantly revert in any other instance through repeated experiments. AFB_1_ from maize treated by Pearl and Blue did appear to become more completely degraded in the second experiment (Table 2). The degradation percentage of samples treated by the Pearl strain in the first run was 88.4% before acid treatment, and 90% after (Table 2). In the second run, degradation percentage changed from 84.1, 84.2, and 94.4% to 80.4, 91.3, and 99% for N001, Pearl and Blue strains, respectively (Table 2).

**Table 2:** Acid reversion assay on breakdown products from AFB_1_-contaminated maize treated by *P. ostreatus*

Experiment no.	Time	Strain		
		N001	Pearl	Blue
1	Before	91.5 ± 2.3^a^	88.4 ± 1.3^a^	94.3 ± 1.0^a^
	After	80.8 ± 3.0^b^	90.0 ± 1.7^a^	88.8 ± 1.3^b^
2	Before	84.1 ± 2.9^a^	84.2 ± 1.3^b^	94.4 ± 0.6^b^
	After	80.4 ± 3.8^a^	91.3 ± 1.7^a^	99.0 ± 0.8^a^

Values represent the mean ± standard error. Values not followed by the same letter within columns of the same experiment are significantly different (*p* < 0.05) by Tukey’s HSD

### Mutagenicity of AFB_1_-contaminated maize treated with *P. ostreatus*

**Fig. 2:**
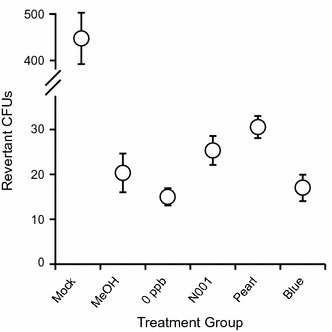
Mutagenicity of breakdown products from AFB_1_-contaminated maize treated with *P. ostreatus.* Number of revertant CFUs when exposed to the extraction solvent without AFB_1_ (MeOH), or extracts from untreated (mock) and treated (N001, Pearl and Blue) maize with initial AFB_1_ concentrations of 2500 ng g^−1^, and from uncontaminated (0 ng g^−1^) maize. *Whiskers* show standard errors

To generate a reference, increasing concentrations of pure AFB_1_ were dissolved in 70% MeOH and the number of *Salmonella* CFUs that reverted from *his* ^−^ to *his* ^+^ increased in a concentration-dependent manner from 0 ng g^−1^ (70% MeOH only) to 10,000 ng g^−1^ (data not shown). Extracts from untreated (mock) samples that had an initial AFB_1_ concentration of 2500 ng g^−1^ resulted in an average of 456 ± 21 revertant CFUs (Fig. 2). Samples treated with N001, Pearl or Blue strains resulted in 26.2 ± 1.9, 29.8 ± 1.7, and 17.2 ± 1.7 revertant CFUs respectively, which were not significantly different than baseline levels from uncontaminated (0 ng g^−1^) maize samples and 70% MeOH controls that resulted in 15 ± 1.9 and 20.3 ± 4.3 revertant CFUs, respectively (Fig. 2).

## Discussion

The purpose of this study was to determine the capacity of the edible, ligninolytic fungus *P. ostreatus* to degrade AFB_1_ in naturally contaminated biomaterial using standard cultivation techniques. The first objective was to measure growth characteristics that are important for *P. ostreatus* production in the presence of increasing concentrations of AFB_1_. Neither vegetative growth, mushroom development nor yield were inhibited by AFB_1_. The second objective was to measure residual AFB_1_ in the substrate after mushroom harvest and test for the presence of AFB_1_ in *P. ostreatus* mushrooms produced from contaminated substrate. Following a standard time course for specialty mushroom production, the data revealed that AFB_1_ was significantly degraded in nearly all treatments. No detectable quantity of aflatoxin could be measured in 100× concentrated extracts from the mushrooms of *P. ostreatus* cultivated on AFB_1_-contaminated maize, regardless of the strain of *P. ostreatus* used or the initial level of AFB_1_ in the initial substrate. The final objective in this work was to evaluate the potential mutagenicity of breakdown products using the *Salmonella* (Ames) mutagenicity assay and by measuring the extent of reversion to the parent compound under conditions simulating the human stomach, both of which were minimal.

Superior degradation of AFB_1_ by the Blue *P. ostreatus* strain used in this study is similar to the findings of Das et al. (2015) who showed that the wild *P. ostreatus* strain GHBBF10 (GenBank accession number KC987361) had greater AFB_1_-degradation capacity than another strain (MTCC 142) from a culture collection. Results from the mutagenicity assay presented here are in agreement with Wang et al. (2011) and Alberts et al. (2009) who used purified manganese peroxidase from *P. sordida*, and purified laccase from another white-rot fungus *Trametes versicolor*, respectively, to degrade AFB_1_ in an ex situ experiment and both found that the mutagenicity of breakdown products were significantly reduced compared to control samples in an enzyme activity-dependent manner. In most cases, the extent that breakdown products reverted to the parent compound in this study were on par with results from the same assay used on breakdown products from AFB_1_-contaminated maize that had been subjected to ammoniation (Weng et al. 1994), the most effective and commonly used chemical decontamination technology. However, breakdown products from AFB_1_-contaminated maize that was treated with the N001 and Blue strains did show significant reversion in the first run of a duplicated acid reversion assay.

*Pleurotus ostreatus* can be cultivated on a diverse array of lignocellulosic substrates that can be directly consumed as, or are byproducts from, food intended for human or animal consumption, and which are also highly susceptible aflatoxin contamination e.g. maize, groundnuts, tree nuts, etc. This work adds to the growing body of evidence showing that microbes, especially white-rot fungi, can be used to degrade aflatoxin in crops intended for livestock consumption (Kim et al. 2017). Livestock also suffer adverse health effects from exposure to AFB_1_ and dairy animals pass a converted form of the toxin through their milk (Kim et al. 2017). The results here show that even highly contaminated maize can be detoxified to levels that are acceptable for some uses as livestock fodder according to U.S. standards. *P.* ostreatus-treated, lignocellulosic materials and edible oyster mushrooms from the genus *Pleurotus* have been used as a feed supplement for a variety of animals in studies unrelated (e.g. Adamovic et al. 1998) and related to aflatoxin contamination including a in a study by Yogeswari et al. (2012) that reported a hepatoprotective effect of *Pleurotus sajor*-*caju* mushrooms on chickens that were simultaneously fed aflatoxin-contaminated feed. Continued research is needed to identify ligninolytic fungal strains that consistently and completely degrade aflatoxin, but the demonstrated degradation capacity of *P. ostreatus* and its renowned edibility make it a superior candidate for further investigation.

## Abbreviations

AFB1: aflatoxin B1
ATCC: American Type Culture Collection
USDA–ARS: United States Department of Agriculture–Agriculture Research Service
MC: moisture content
MeOH: methanol
BE: bioefficiency
ANOVA: analysis of variance
HSD: honest significant difference
